# The effectiveness of social support interventions on loneliness among older people in the community: a meta-analysis of randomised controlled trials

**DOI:** 10.3389/fragi.2025.1594513

**Published:** 2026-01-07

**Authors:** Guangting Huang, Xiaotong Yang, Li Yao, Xiaofang Li, Yuanping Wu, Shiqi Zhou, Yinhua Wang

**Affiliations:** 1 The Affiliated Hospital of Guizhou Medical University, Guiyang, China; 2 Guizhou Medical University, Guiyang, Guizhou, China; 3 Weng’an County People’s Hospital, Weng’an, Guizhou, China

**Keywords:** social support, loneliness, older adults, community dwelling, meta-analysis

## Abstract

**Background:**

To combat loneliness among elderly individuals, it is crucial to identify effective strategies that can alleviate the negative impact of loneliness on their overall well-being.

**Objective:**

This study evaluated social support programs’ impact on loneliness in community-dwelling older adults. The goal was to inform tailored interventions that decrease loneliness and improve quality of life.

**Methods:**

We systematically searched ten electronic databases (EMBASE, PubMed, Cochrane Library, Web of Science, CNKI, Weipu, WanFang, CBM) from inception to December 31, 2023, with citation chasing. Included randomized controlled trials (RCTs) tested social support interventions for loneliness reduction. Two independent reviewers extracted participant details, study characteristics, interventions, and outcomes. The methodological rigor of the included studies was assessed by JBI critical appraisal checklists.

**Results:**

Nineteen studies met inclusion criteria, with over half from China (n = 7) and the United States (n = 4). Meta-analysis showed that social support helped alleviate loneliness and the difference was statistically significant [SMD = −0.60, 95%*CI* (−1.00, −0.20), *I* ^2^ = 93%, *P* = 0.003, random effect model]. Subgroup analysis showed significantly lower loneliness scores in experimental groups at less than 3 months [SMD = −0.68, 95%*CI* (−1.31, −0.06), *I* ^2^ = 93%, *P* = 0.03, random effect model]. In addition, multiple-intervention groups also showed significantly lower scores versus controls. The combined result was [SMD = −1.26, 95%*CI* (−2.20, −0.32), *I* ^2^ = 97%, *P* = 0.008, random effect model].

**Conclusion:**

Social support interventions effectively reduce loneliness among community-dwelling older adults. For practical application, community health professionals are encouraged to implement short-term (≤3 months) multicomponent programs that combine emotional, peer, and technological support, delivered through group counseling, tele-support, or structured social activities. Integrating these interventions into routine community nursing services and local age-friendly programs may enhance mental wellbeing and social connectedness among older adults.

## Introduction

Loneliness is described as an involuntary social isolation, which is prevalent among older adults and may limit their access and ability to maintain social relationships (Lampinen et al., 2022; Manzanares et al., 2021). Several studies have indicated that more than 33% of this population experiences isolation (Gardiner et al., 2020; Timmermans et al., 2019). Moreover, the frequency of loneliness rises with age (Chen and Gong, 2022). This condition significantly impacts physical and mental health, being linked to immune dysfunction, inflammation, cognitive decline, depression, anxiety, hypertension, and even premature mortality (Lapane et al., 2022; Yu et al., 2021; Perissinotto et al., 2019). Hence, identifying effective strategies to reduce loneliness among community-dwelling older adults has become a critical public health priority.

Social isolation refers to an objective lack of social contact, whereas loneliness is a subjective perception of insufficient or unsatisfying social relationships (de Jong-Gierveld et al., 2006; Petersen et al., 2020). This distinction is particularly important in the present study, because prior research often combined these two outcomes, leading to inconsistent findings regarding intervention effectiveness. By focusing specifically on loneliness—a subjective emotional experience—our study provides more precise evidence to guide the design of interventions that target perceived rather than structural deficits in social relationships.

Social support has been identified as a key protective factor against loneliness, encompassing emotional support (expressions of empathy and care), instrumental or practical support (assistance with daily activities), and informational support (advice and guidance) (Kassim and Ahmad Badayai, 2023). Zhang demonstrated a negative correlation between higher social support levels and loneliness (Zhang and Dong, 2022). Conversely, inadequate social connectedness increases loneliness risk in older adults (O'Rourke et al., 2018). Some research suggests that having a large social network may be beneficial, the quality of relationships within that network, such as feeling understood, valued, and supported, may be more critical in reducing loneliness (Hawkley and Kocherginsky, 2018). While short-term support interventions show positive effects (Dahlberg et al., 2022), questions remain regarding which forms and durations of support yield the most sustainable effects. Some researchers have previously systematically reviewed interventions for social isolation, which included objective social isolation and loneliness (Ibarra et al., 2020; Ma et al., 2020; Noone et al., 2020). Such an approach may obscure the specific effectiveness of social support on loneliness itself. Hence, our study addressed important evidence gaps in existing literature by: Isolating social support as a standalone intervention category; including only community-based studies to improve contextual relevance; conducting detailed subgroup analyses on duration and format of interventions. These refinements are crucial for informing targeted, sustainable, and culturally appropriate interventions that can be scaled within community health systems.

To address these gaps, this study aimed to analyze randomized controlled trials and perform a meta-analysis to assess the impact of social support interventions on loneliness in older adults living in the community. Our approach provides a more targeted examination of a single social support intervention type, across culturally diverse settings including China, which has been underrepresented in prior meta-analyses. Moreover, we explore effect moderators such as intervention duration (<3 months, 3–6 months, >6 months) and intervention complexity (single and multicomponent). Developing specific and efficient social support programs can aid in decreasing feelings of isolation and enhancing the overall quality of life for older individuals living in the community.

## Methods

The guidelines outlined in the PRISMA Extension Statement for Reporting of Systematic Reviews that Include Meta-Analyses of Health Care Interventions were adhered to when reporting this meta-analysis (Hutton et al., 2015). The research has been properly recorded in PROSPERO with the registration code CRD42020226523. An earlier version of the study protocol has been published (Wang et al., 2023).

### Search strategy

A comprehensive systematic search was conducted across eight electronic databases from inception to 30 June 2024. The following databases were queried: EMBASE, PubMed, the Cochrane Library, Web of Science, China National Knowledge Infrastructure (CNKI), China Science and Technology Journal Database (Weipu), WanFang Database, and China Biology Medicine disc (CBM). Search terms were developed by combining Medical Subject Headings (MeSH) and free-text keywords related to three domains: loneliness (e.g., “social isolation” or “loneliness” or “social exclusion” or “social exclusions” or “ostracism” or “social alienation”), old adults (e.g., “aged” or “elderly” or “oldest old” or “agenarians” or “nonagenarian” or “octogenarian” or “centenarian” or “old people or “old person” or “old adult”), and social support interventions (e.g., “social support” or “social care” or “psychosocial support” or “psychological support”). Citation chasing was performed by manually screening the reference lists of all included articles and relevant reviews to identify additional studies. Only studies published in English and Chinese were included, due to database coverage and the language proficiency of the review team. Grey literature was not included, as this study focused on peer-reviewed evidence.

### Inclusion and exclusion criteria

Inclusion criteria for the studies were as follows: (a) individuals aged 60 years or older residing in the community; (b) the experimental group received social support interventions of any kind; (c) validated tools like the Los Angeles Loneliness Scale from UCLA (UCLA), the De Jong Loneliness Scale (DJLS), the PROMIS-L Social Isolation Scale (PROMIS-L), Ando-Osada-Kodama (AOK) loneliness scale; (d) comparisons were made against passive (usual care) or active (non-social support) control groups; (e) study design was randomized controlled trial (RCT); (f) publications were in English or Chinese. The exclusion criteria were (a) no full text; (b) studies that reported duplicate data.

### Study selection and data extraction

The research selection and data extraction process involved three reviewers. Two reviewers autonomously chose all research papers based on their titles, abstracts, and full texts, following the specified inclusion and exclusion criteria. To address any inconsistencies or disputes that arose during the selection process, a third reviewer was brought in for consultation.

Data were extracted using astandardized form. Included in the data were basic information, participant details (including number, eligibility, age, and gender), intervention/exposure specifics (type, frequency, duration, content, comparison with control group, delivery format, provider information), outcome data (effect size, standard deviations, statistical significance), and instruments used. Data missing for relevant outcomes were obtained from original authors via email.

### Quality assessment of included studies

Two researchers independently evaluated the methodological rigor of the studies included, utilizing the Joanna Briggs Institute (JBI) critical appraisal checklists for randomized controlled trials (RCTs) (Munn et al., 2020). The checklist for randomized controlled trials includes 13 items that can be answered with “yes,” “no,” “unclear” or “not applicable,” and a “yes” response is worth one point. Referring to the JBI manual and previous research, RCTs with scores less than seven points were identified as weak quality studies. Any disagreements among reviewers were settled through consensus or conversation with a third reviewer.

### Statistical analysis

Review Manager (RevMan 5.3) and R Studio softwares were used for quantitative analysis of selected studies. The research utilized a narrative approach that was not suitable for quantitative analysis in the meta-analysis. The inverse variance method was employed in meta-analysis to determine the weights assigned to the studies. Effect size was determined by calculating the mean and standard deviation (SD) of the difference between scores at the last follow-up and baseline. In the pooled analysis, mean differences (MD) or standardized mean differences (SMD) were utilized as effect estimates along with their corresponding 95% confidence intervals (CI). SMD is utilized when assessing the identical result using a distinct tool compared to the identical result assessed using the identical tool. Statistical heterogeneity was assessed using the Chi-squared test and I^2^ statistic. A random-effects model was adopted when significant heterogeneity was detected (I^2^ > 40% or p < 0.05); otherwise, a fixed-effects model was used. Sensitivity analyses were conducted by excluding lower-quality studies to examine the robustness of results. Sensitivity tests were performed to evaluate the impact of a low-quality study on combined results. Publication bias was evaluated visually via funnel plots and statistically using Egger’s regression test. An analysis of subgroups was conducted to investigate how various lengths and forms of social support impact results.

## Results

### Study selection

**FIGURE 1:**
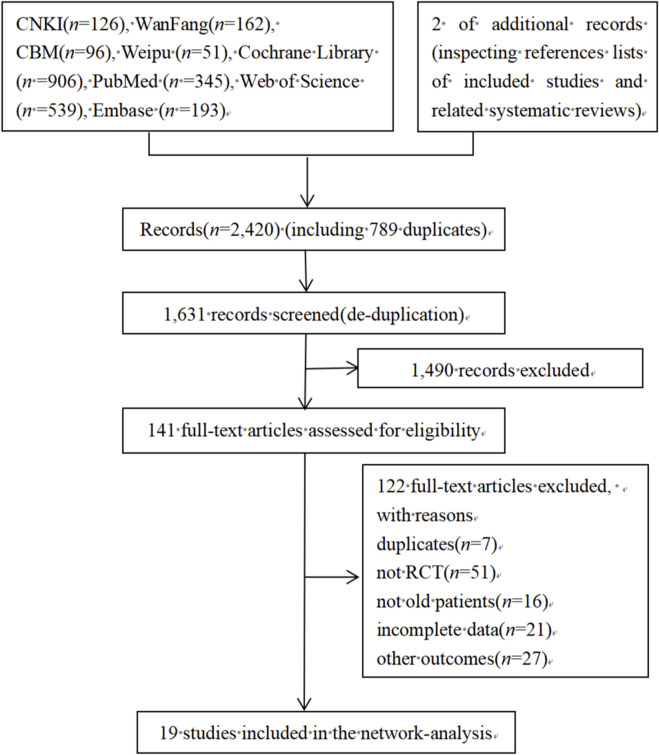
PRISMA flow chart of study selection.

A total of 2,420 articles were retrieved in Figure 1. Out of the articles reviewed, 789 duplicates were removed using Endnote software, 1,490 articles were eliminated based on title and abstract, leaving 141 articles for full-text screening. This study ultimately incorporated 19 research studies (Banks and Banks, 2005; Chang et al., 2018; Choi et al., 2020; Cohen-Mansfield et al., 2018; Galinha et al., 2021; Gilbody et al., 2021; Gu et al., 2019; José. et al., 2022; Ilgaz et al., 2023; Kremers et al., 2006; Lai et al., 2020; Larsson et al., 2016; Ristolainen et al., 2020; Rodríguez-Romero et al., 2021; Saito et al., 2012; Wang et al., 2014; Wei et al., 2021; Yang et al., 2023; Zhou et al., 2014).

### Study characteristics

The characteristics of the included studies are displayed in Table 1. China (*n* = 7) and United States (*n* = 4) accounted for the majority of the studies conducted. The 16 studies included 1,809 subjects, with sample sizes varying from 14 to 392 older adults. The average age of the population fell between 60.00 and 84.17 years. The types of interventions were divided into single intervention (*n* = 12) and multiple intervention (*n* = 7). The single intervention were various, including group counseling (Gu et al., 2019; Wang et al., 2014), tele-support (Choi et al., 2020; Gilbody et al., 2021), social activity (Galinha et al., 2021; Larsson et al., 2016), animal-assisted therapy (Banks and Banks, 2005), professional support (Zhou et al., 2014), peer support (Lai et al., 2020), Group intervention of Technology (Wei et al., 2021), support from themselves (Kremers et al., 2006), and I-SOCIAL intervention (Cohen-Mansfield et al., 2018). The interventions in control group were usual care (*n* = 12), follow-up visits (*n* = 2), no treatment (*n* = 2), and tele-support (*n* = 3). The intervention duration ranged from 4 weeks to 3 years. Half of the studies conducted follow-up after intervention, but other studies did not describe the follow-up period. Follow-up duration varied between 1 week and 6 months. The level of loneliness was assessed through various scales including UCLA (*n* = 12), DJLS (*n* = 4), PROMIS-L (*n* = 1), AOK loneliness scale (*n* = 1), and loneliness scale for the elderly (*n* = 1). There are numerous variations of the instruments, with UCLA having five versions and DJLS having two.

**TABLE 1:** Study and patient characteristics (*n* = 19).

Author (year)	Country	Age(E.G.,/CG)	Gender(M/F)	Sample size	Type of intervention	Type of control	Duration of trial	Follow up	Instruments
Ayşegül and Sebahat (2023)	Turkey	≥65	0/69	69	Group support, person-centred interventions	Usual care	12 weeks	NA	Loneliness scale for the elderly
Banks and Banks (2005)	United States	83.2 ± 5.4/81.1 ± 4.5	14/19	33	Animal-assisted therapy	Active control	6 weeks	NA	UCLA scale 20 item
Chang et al. (2018)	China	80.56 ± 4.98/80.47 ± 4.53	44/55	99	Theory-based multi-level interventions	Usual care	6 months	NA	UCLA scale 20 item
Choi et al. (2020)	United States	74.4 ± 8.2/73.5 ± 9.8	34/55	89	Tele-support	Tele- support (friendly visits and active control by phone)	5 weeks	6–12 weeks	Patient-reported outcomes measurement information system) social isolation scale (PROMIS-L)
Cohen-Mansfield et al. (2018)	United States	76.6 ± 6.8/79 ± 6.62	14/60	74	I-SOCIAL intervention	Usual care	3 years	3 months	UCLA scale 8 item
Larsson et al. (2016)	Sweden	>60	6/24	30	Social activity	Usual care	3 months	3 months	UCLA scale 20 item
Galinha et al. (2021)	United States	76.76 ± 8.93/76.51 ± 8.66	25/124	149	Social activity	Usual care	4 months	NA	UCLA scale 4 item
Gilbody et al. (2021)	North East of England	74.2 ± 5.4/74.1 ± 5.6	37/59	96	Tele support	Usual care	4–6 weeks	1–3 months	De Jong loneliness scale 11-item
Gu et al. (2019)	China	83.42 ± 4.48/84.17 ± 5.22	21/23	44	Group counseling	Usual care	4 weeks	NA	UCLA scale 20 item
José et al. (2022)	Spain	80.79 ± 5.38/82.91 ± 6.86	28/91	119	Multicomponent support	Usual care	16 weeks	6 months	De Jong loneliness scale 6-item
Kremers et al. (2006)	Netherlands	62.8 ± 6.4/65.2 ± 7.6	0/119	119	Support from themselves	No treatment	6 weeks	6 months	De Jong loneliness scale 11-item
Lai et al. (2020)	China	>60	22/38	60	Peer support	Tele- support (Phone)	8 weeks	1–10 weeks	De Jong loneliness scale 6-item
Ristolainen et al. (2020)	Finland	76.8 ± 7.2/76.8 ± 7.76	67/325	392	Social support, Counseling, and activities	Usual care	6 months	3–6 months	UCLA scale 12-item
Rodríguez-Romero et al. (2021)	Barcelona	80.5 ± 6.6/79.8 ± 17.4	29/26	55	Community and professional support	Usual care	6 months	NA	UCLA scale
Saito et al. (2012)	Japan	72.6 ± 4.4/72.8 ± 4.8	20/40	60	A group-based educational, cognitive, and social support program	Usual care	1 month	1–6 months	Ando-Osada-Kodama (AOK) loneliness scale
Wang et al. (2014)	China	>60	NA	30	Group counseling	Usual care	4 weeks	NA	UCLA scale 20 item
Wei et al. (2021)	China	>60	NA	14	Group intervention of technology	No treatment	5 weeks	NA	ULS scale 6 item
Yang et al. (2023)	China	68.07 ± 6.68/69.00 ± 6.04	32/57	89	Tele-support and peer support	Tele-support (line group)	8 weeks	NA	UCLA scale 20 item
Zhou et al. (2014)	China	>60	94/114	208	Professional support	Follow-up visits	1 year	NA	UCLA scale 20 item

NR: Not Reported.

### Methodological quality and risk of bias within studies

The summary of the bias risk in the 19 studies was provided in supplementary material. 17 RCTs met 7–13 items. No randomized controlled trials achieved complete blinding of participants, providers, and evaluators at the same time. Five randomized controlled trials failed to meet the five criteria of the JBI critical appraisal checklist. Twelve randomized controlled trials did not clearly report allocation concealment, did not conduct intention-to-treat analysis, and did not blind participants, implementers, and outcome assessors.

### Effect of social support on reducing loneliness

**FIGURE 2:**
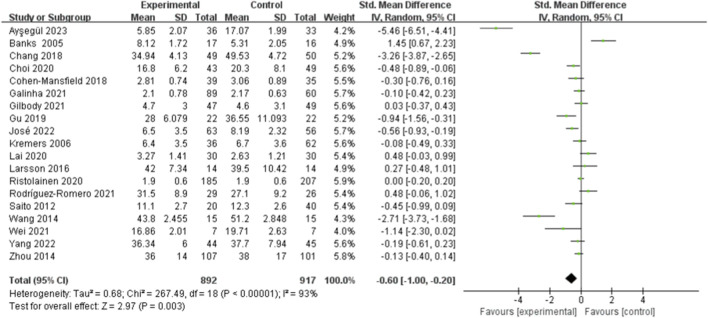
Effect of social support on loneliness in older adults.

A meta-analysis was performed on 19 studies in Figure 2 to investigate how social support can reduce loneliness. The analysis included 1809 participants, with 892 in the experimental group and 917 in the control group. The primary analysis showed a significant impact of the intervention compared to the control group in decreasing feelings of isolation [SMD = −0.60, 95%*CI* (−1.00, −0.20), *P* = 0.003, random effect model], suggesting a substantial effect with a wide yet meaningful confidence range. The studies showed a significant amount of diversity (*I* ^2^ = 93%, *P* < 0.001), suggesting a substantial level of variation among them. Therefore, further subgroup analysis was conducted to examine potential factors explaining the heterogeneity among these studies.

**FIGURE 3:**
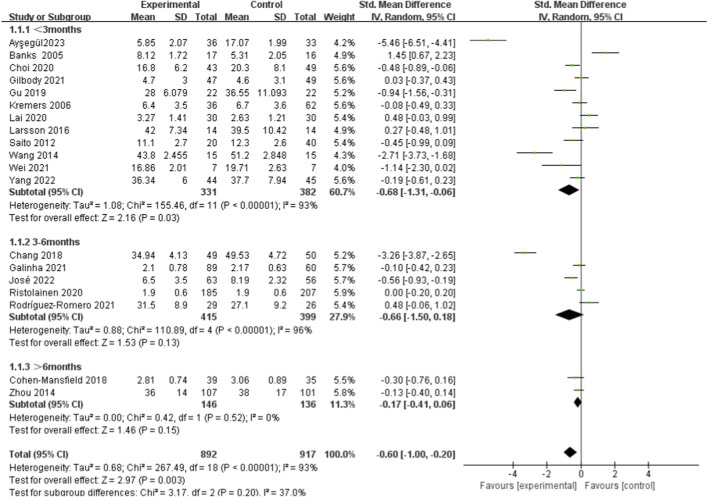
Subgroup analysis of different duration of social support on loneliness in older adults.

Given the potential impact of intervention duration on effectiveness, this study categorized duration into three subgroups. The findings indicated that participants in the experimental group experienced notably reduced feelings of lonely in comparison to those in the control group within less than 3 months. The combined results were [SMD = −0.68, 95%*CI* (−1.31, −0.06), *I* ^2^ = 93%, *P* = 0.03, random effect model]. No significant variances were noted between the intervention and control groups when the intervention lasted 3–6 months or more than 6 months [SMD = −0.66, 95%*CI* (−1.50, 0.18), *I* ^2^ = 96%, *P* = 0.13, random effect model] and [SMD = −0.17, 95%*CI* (−0.41, 0.06), *I* ^2^ = 0%, *P* = 0.52, random effect model] in Figure 3.

**FIGURE 4:**
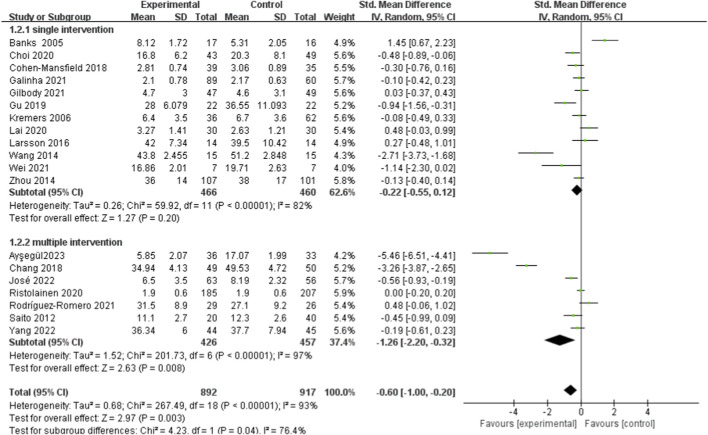
Subgroup analysis of different types of social support on loneliness in older adults.

It stratified interventions into two subgroups based on the potential impact of intervention types on efficacy, including single intervention and multiple intervention. The findings indicated that the multiple intervention group had notably reduced feelings of loneliness in comparison to the control group. The pooled results were [SMD = −1.26, 95%*CI* (−2.20, −0.32), *I* ^2^ = 97%, *P* < 0.001, random effect model]. In contrast, the subset that received single treatment did not display a notable distinction between the groups that received intervention and those that did not [SMD = −0.22, 95%CI (−0.55, 0.12), I^2^ = 82%, P = 0.200, random effect model] in Figure 4.

### Publication bias analysis

**FIGURE 5:**
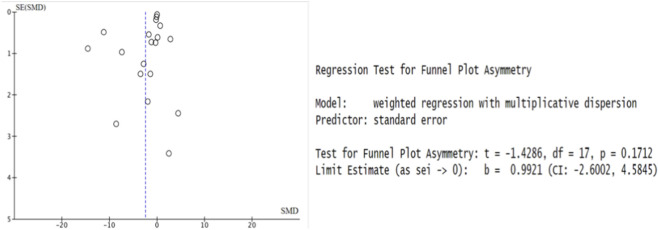
Funnel plot and Egger test results.

According to the funnel plot analysis and the Egger test conducted using R Studio on 19 included papers in this study (t = −1.43, df = 17, p = 0.171), suggesting low probability of publication bias in Table 2 and Figure 5.

**TABLE 2:** Reporting quality assessment of 19 publications (JBI critical appraisal checklist for randomized controlled trials).

Author, year/items	Q1		Q2		Q3		Q4		Q5		Q6		Q7		Q8		Q9		Q10		Q11		Q12		Q13	
	R1	R2	R1	R2	R1	R2	R1	R2	R1	R2	R1	R2	R1	R2	R1	R2	R1	R2	R1	R2	R1	R2	R1	R2	R1	R2
Ayşegül, 2023	Y	Y	UN	UN	Y	Y	N	N	UN	UN	UN	UN	Y	Y	Y	Y	Y	Y	Y	Y	Y	Y	Y	Y	Y	Y
Banks and Banks (2005)	UN	UN	UN	UN	Y	Y	N	N	N	N	N	N	Y	Y	Y	Y	Y	Y	Y	Y	Y	Y	Y	Y	Y	Y
Chang et al. (2018)	Y	Y	UN	UN	Y	Y	N	N	N	N	UN	UN	Y	Y	Y	Y	Y	Y	Y	Y	Y	Y	Y	Y	Y	Y
Choi et al. (2020)	Y	Y	UN	UN	Y	Y	N	N	N	N	N	N	Y	Y	Y	Y	N	N	Y	Y	Y	Y	Y	Y	Y	Y
Cohen-Mansfield et al. (2018)	UN	UN	UN	UN	Y	Y	N	N	N	N	N	N	Y	Y	Y	Y	Y	Y	Y	Y	Y	Y	Y	Y	Y	Y
Larsson et al. (2016)	Y	Y	Y	Y	Y	Y	N	N	N	N	Y	Y	Y	Y	Y	Y	Y	Y	Y	Y	Y	Y	Y	Y	Y	Y
Galinha et al. (2021)	Y	Y	Y	Y	Y	Y	N	N	N	N	Y	Y	Y	Y	Y	Y	Y	Y	Y	Y	Y	Y	Y	Y	Y	Y
Gilbody et al. (2021)	Y	Y	Y	Y	N	N	N	N	N	N	Y	Y	Y	Y	N	N	N	N	Y	Y	Y	Y	Y	Y	Y	Y
Gu et al. (2019)	Y	Y	UN	UN	Y	Y	N	N	N	N	UN	UN	Y	Y	Y	Y	Y	Y	Y	Y	Y	Y	Y	Y	Y	Y
José, 2022	Y	Y	UN	UN	Y	Y	N	N	N	N	UN	UN	N	N	Y	Y	Y	Y	Y	Y	Y	Y	Y	Y	Y	Y
Kremers et al. (2006)	Y	Y	UN	UN	Y	Y	N	N	N	N	UN	UN	Y	Y	Y	Y	Y	Y	Y	Y	Y	Y	Y	Y	Y	Y
Lai et al. (2020)	Y	Y	Y	Y	N	N	Y	Y	Y	Y	N	N	Y	Y	Y	Y	Y	Y	Y	Y	Y	Y	Y	Y	Y	Y
Ristolainen et al. (2020)	Y	Y	UN	UN	Y	Y	N	N	N	N	UN	UN	Y	Y	Y	Y	Y	Y	Y	Y	Y	Y	Y	Y	Y	Y
Rodríguez-Romero et al. (2021)	Y	Y	UN	UN	Y	Y	N	N	N	N	N	N	Y	Y	Y	Y	Y	Y	Y	Y	Y	Y	Y	Y	Y	Y
Saito et al. (2012)	Y	Y	N	N	N	N	N	N	N	N	N	N	Y	Y	Y	Y	Y	Y	Y	Y	Y	Y	Y	Y	Y	Y
Wang et al. (2014)	Y	Y	UN	UN	Y	Y	N	N	N	N	UN	UN	Y	Y	Y	Y	Y	Y	Y	Y	Y	Y	Y	Y	Y	Y
Wei et al. (2021)	UN	UN	UN	UN	Y	Y	N	N	N	N	UN	UN	Y	Y	Y	Y	Y	Y	Y	Y	Y	Y	Y	Y	Y	Y
Yang et al. (2023)	Y	Y	UN	UN	Y	Y	Y	Y	UN	UN	UN	UN	Y	Y	Y	Y	Y	Y	Y	Y	Y	Y	Y	Y	Y	Y
Zhou et al. (2014)	Y	Y	UN	UN	Y	Y	N	N	N	N	UN	UN	Y	Y	Y	Y	Y	Y	Y	Y	Y	Y	Y	Y	Y	Y

N, No; Y, Yes; UN, Unclear; R1, Rater #1; R2, Rater #2. Q1, Was a truly randomised grouping of study participants used? Q2, Was allocation to treatment groups concealed? Q3, Were treatment groups similar at the baseline? Q4, Were participants blind to treatment assignment? Q5, Were those delivering treatment blind to treatment assignment? Q6, Were outcomes assessors blind to treatment assignment? Q7, Were treatment groups treated identically other than the intervention of interest? Q8, Was follow up complete and if not, were differences between groups in terms of their follow up adequately described and analyzed? Q9, Were participants analyzed in the groups to which they were randomized? Q10, Were outcomes measured in the same way for treatment groups? Q11, Were outcomes measured in a reliable way? Q12, Was appropriate statistical analysis used? Q13, Was the trial design appropriate, and any deviations from the standard RCT, design (individual randomization, parallel groups) accounted for in the conduct and analysis of the trial?

## Discussion

This meta-analysis provides the first comprehensive evaluation of interventions aimed at providing social support to address loneliness among older individuals. In this study, we meta-analyzed data from 19 peer-reviewed articles and involved 1809 participants. We found a significant result about social support on loneliness, which was differ from some studies. According to Ma, R. M., systematic evaluations of interventions on loneliness and social isolation did not show clear effects (Ma et al., 2020). The notable finding in this research could be associated with the particular strategy known as social support. This study verified that social support can be considered a useful healthcare service to reduce loneliness in elderly.

Social support encompasses various forms of aid such as tangible help, emotional support, close relationships, guidance, input, and beneficial social connections within different areas of support (Wang et al., 2023). According to the different sources of social support, which includes community support, volunteer support, family support, professional support, and peer support (Chen et al., 2014; Zhang and Dong, 2022). There is difficulty in conducting a subgroup analysis of social support based on the intervention classification in the included studies. Hence, we divided the types of interventions into single intervention and multiple intervention. The results indicate that multiple interventions are more effective than single interventions. It is recommended that researchers should develop rigorous intervention program based on the classification of social support, to provide clear guidance for clinical nursing staff.

Group counseling, tele-support and social activity were the popular types of social support. Providing individuals with a shared space where they can express their feelings of loneliness and be understood by others experiencing similar emotions and professional development is proven to be beneficial in group counseling (Gu et al., 2019). Tele-support plays several roles in addressing loneliness and provides a convenient platform for individuals to establish and maintain social connections remotely. It can provide remote mental health support, educational programs, crisis intervention for old people (Bar-Tur et al., 2022; Choi et al., 2020). Additionally, it demonstrated that engaging in social activities fosters emotional support by fostering a sense of belonging to a group. The identification with a group is essential for older adults to experience wellbeing and health advantages (Galinha et al., 2021). Tele-support and social activities constitute essential components of standard community care for older adults, given their demonstrable efficacy and broad applicability.

According to Dahlberg’s research identified that interventions focusing on social connectedness had short-term but not sustained effects on loneliness (Dahlberg et al., 2022), our subgroup analysis highlighted short-term (<3 months) interventions as optimal for effectiveness and feasibility. Balancing a sustainability threshold (<6 months) that mitigates attrition risks (Tkatch et al., 2021; Bruce et al., 2021). Prolonged interventions demonstrate diminishing returns due to participant fatigue (Fakoya et al., 2020), reduced relevance from evolving health/social circumstances (Jin et al., 2021), and resource inefficiency (Quan et al., 2020). This equilibrium period maximizes intervention efficacy while preventing the diminished benefits observed in longer trials.

### Strengths and limitations

There are multiple advantages to this research. This study explored the effects of different social support on elderly loneliness. Analyzing various durations and types of interventions further assessed the impact of social support on loneliness in elderly individuals. Additionally, the present research has its own constraints. Due to inconsistent classification of social support in the included studies is confused, it is not possible to analyze subgroup based on a certain classification and determine which specific type of social support is more effective for elderly loneliness. Although the included studies provided valuable insight, some contextual factors such as gender, socioeconomic status, and rural–urban differences were rarely reported or inconsistently measured, precluding subgroup analysis. The instruments of outcomes included in the study were validated tools, there were too many versions available. It may contribute to statistical heterogeneity. Despite efforts to reach out to the authors, numerous studies were excluded during the literature screening process due to insufficient data. This may lead to a potential bias about our results. Variations in national healthcare systems and community care structures may influence the implementation and sustainability of social support interventions. Additionanlly, differences in intervention providers can lead to inconsistencies in quality and intensity, contributing to heterogeneity. Future research should further examine how these factors affect intervention outcomes.
