# Supplementary material for: The effectiveness of social support interventions on loneliness among older people in the community: a meta-analysis of randomised controlled trials
Source: Front Aging. 2026 Jan 7;6:1594513. doi: 10.3389/fragi.2025.1594513 (PMC12819595; doi:10.3389/fragi.2025.1594513)
Supplement: Supplementary file 1 [file Table1.docx]

| **English databases retrieval strategies** | | |
| --- | --- | --- |
| PubMed | #1 | social isolation OR loneliness OR social exclusion* OR ostracism OR social alienation |
|  | #2 | aged OR elderly OR oldest old OR agenarian* OR nonagenarian* OR octogenarian* OR centenarian* OR old people OR old person* OR old adult* |
|  | #3 | social support OR social care OR psychosocial support OR psychological support |
|  | #4 | #1 AND #2 AND #3 |
|  | *n*=345 | |
| Embase | #1 | social isolation OR loneliness OR social exclusion OR social exclusions OR ostracism OR social alienation |
|  | #2 | aged OR elderly OR oldest old OR agenarian OR agenarians OR nonagenarian OR nonagenarians OR octogenarian OR octogenarians OR centenarian OR centenarians OR old people OR old person OR old persons OR old adult OR old adults |
|  | #3 | social support OR social care OR psychosocial support OR psychological support |
|  | #4 | #1 AND #2 AND #3 |
|  | *n*=193 | |
| Cochrane Library | #1 | social isolation OR loneliness OR social exclusion* OR ostracism OR social alienation |
|  | #2 | aged OR elderly OR oldest old OR agenarian* OR nonagenarian* OR octogenarian* OR centenarian* OR old people OR old person* OR old adult* |
|  | #3 | social support OR social care OR psychosocial support OR psychological support |
|  | #4 | #1 AND #2 AND #3 |
|  | *n*=906 | |
| Web of Science | #1 | social isolation OR loneliness OR social exclusion OR social exclusions OR ostracism OR social alienation |
|  | #2 | aged OR elderly OR oldest old OR agenarian OR agenarians OR nonagenarian OR nonagenarians OR octogenarian OR octogenarians OR centenarian OR centenarians OR old people OR old person OR old persons OR old adult OR old adults |
|  | #3 | social support OR social care OR psychosocial support OR psychological support |
|  | #4 | #1 AND #2 AND #3 |
|  | *n*=539 | |
| **Chinese databases retrieval strategies** | | |
| China National Knowledge Infrastructure (CNKI) | #1 | 孤独感 OR 孤独心理 OR社会隔离 OR 社会孤立 OR 社交孤独 OR 社交孤立 |
|  | #2 | 老年人 OR 老人 OR 老年人群 |
|  | #3 | 社会支持 OR 社会保障 OR 社会支援 OR 心理支持 OR 社会优抚 |
|  | #4 | #1 AND #2 AND #3 |
|  | *n*=126 | |
| China Science and Technology Journal Database (Weipu) | #1 | 孤独感 OR 孤独心理 OR社会隔离 OR 社会孤立 OR 社交孤独 OR 社交孤立 |
|  | #2 | 老年人 OR 老人 OR 老年人群 |
|  | #3 | 社会支持 OR 社会保障 OR 社会支援 OR 心理支持 OR 社会优抚 |
|  | #4 | #1 AND #2 AND #3 |
|  | *n*=51 | |
| WanFang Database | #1 | 孤独感 OR 孤独心理 OR社会隔离 OR 社会孤立 OR 社交孤独 OR 社交孤立 |
|  | #2 | 老年人 OR 老人 OR 老年人群 |
|  | #3 | 社会支持 OR 社会保障 OR 社会支援 OR 心理支持 OR 社会优抚 |
|  | #4 | #1 AND #2 AND #3 |
|  | *n*=162 | |
| China Biology Medicine disc (CBM) | #1 | 孤独感 OR 孤独心理 OR社会隔离 OR 社会孤立 OR 社交孤独 OR 社交孤立 |
|  | #2 | 老年人 OR 老人 OR 老年人群 |
|  | #3 | 社会支持 OR 社会保障 OR 社会支援 OR 心理支持 OR 社会优抚 |
|  | #4 | #1 AND #2 AND #3 |
|  | *n*=96 | |
