# Supplementary figures and images for: The effectiveness of social support interventions on loneliness among older people in the community: a meta-analysis of randomised controlled trials
Source: Front Aging. 2026 Jan 7;6:1594513. doi: 10.3389/fragi.2025.1594513 (PMC12819595; doi:10.3389/fragi.2025.1594513)

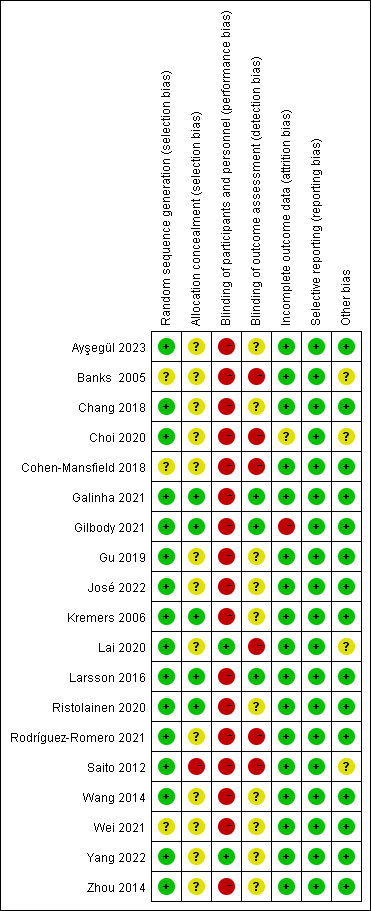

Supplement: Supplementary file 2 [file Image1.tif]
